# What’s Happening on the Other Side? Revealing Nano-Meter Scale Features of Mammalian Cells on Engineered Textured Tantalum Surfaces

**DOI:** 10.3390/ma12010114

**Published:** 2018-12-31

**Authors:** Ting Y. Tsui, Megan Logan, Hassan I. Moussa, Marc G. Aucoin

**Affiliations:** 1 Department of Chemical Engineering, University of Waterloo, Waterloo, ON N2L 3G1, Canada; m3logan@uwaterloo.ca (M.L.); h2moussa@uwaterloo.ca (H.I.M.); marc.aucoin@uwaterloo.ca (M.G.A.); 2 Waterloo Institute of Nanotechnology, University of Waterloo, Waterloo, ON N2L 3G1, Canada

**Keywords:** tantalum, mammalian cells, morphology, adhesion, cross-sectioning, nanoscale

## Abstract

Advanced engineered surfaces can be used to direct cell behavior. These behaviors are typically characterized using either optical, atomic force, confocal, or electron microscopy; however, most microscopic techniques are generally restricted to observing what’s happening on the “top” side or even the interior of the cell. Our group has focused on engineered surfaces typically reserved for microelectronics as potential surfaces to control cell behavior. These devices allow the exploration of novel substrates including titanium, tungsten, and tantalum intermixed with silicon oxide. Furthermore, these devices allow the exploration of the intricate patterning of surface materials and surface geometries i.e., trenches. Here we present two important advancements in our research: (1) the ability to split a fixed cell through the nucleus using an inexpensive three-point bend micro-cleaving technique and image 3D nanometer scale cellular components using high-resolution scanning electron microscopy; and (2) the observation of nanometer projections from the underbelly of a cell as it sits on top of patterned trenches on our devices. This application of a 3-point cleaving technique to visualize the underbelly of the cell is allowing a new understanding of how cells descend into surface cavities and is providing a new insight on cell migration mechanisms.

## 1. Introduction

Cell function, adhesion behavior, and morphology are often influenced by their micro-environments [1,2,3,4,5,6,7,8,9,10,11,12,13,14]. When cells adhere to a surface, this micro-environment is highly influenced by the surface itself. Some of the important characteristics of the surface include, but are not limited to, their mechanical properties (i.e., elastic modulus, pattern geometry), chemical potential, and their ability to interact with other materials in the environment (i.e., adsorb proteins from solutions). This knowledge provides researchers and medical device manufacturers with new tools to control how cells interact with materials [15,16,17].

To understand the mechanisms that drive cell behavior on engineered surfaces, researchers often visually inspect cell surface morphology. However, it could be argued that some of the most important information in determining cell behavior is located on the underbelly of the cell, where the cell meets the substrate [10,18,19]. Are cells that have been observed to float on top of dense pillar patterns [10,18,19,20] or narrowly spaced line structures [21] truly floating? It is known that on widely spaced topographic features, cells wrap around the features [1,13,20,22] thus maximizing their contact. Questions about the cell underbelly span many different applications including fundamental cell research [10,19,20,23,24,25,26], tissue engineering [1,22], surgical implant surface design [27,28,29], and cell immobilization [20,30]. While the physical interaction of the cell and the surface can be visualized near the cell’s periphery by sample tilting, information about the physical interaction between the underbelly of the cell, and the surface is lacking. Furthermore, given the heterogeneity in the composition of the cell, the entire cell should not be expected to have the same interaction with the surface i.e., does the nucleus play a role in how the cell conforms to surface structures? Sub-cellular structures are composed of different materials and have different mechanical properties. Differentiated cell nuclei are 5–10 times stiffer than the cytoskeleton [31]. Callile et al [32] showed the elastic modulus of an endothelial cell nucleus and cytoplasm were 8 and 0.5 kPa, respectively. Antonacci and Braakman [33] measured the longitudinal moduli for the nucleolus, nuclear envelope, and cytoplasm of endothelial cells using Brillouin microscopy and reported that the nucleolus has the largest modulus of the three. Hence, the nucleolus is expected to be the least conforming part of a cell. Unfortunately, there are only a few studies that demonstrate how these sub-cellular organelles may affect the cell morphology on patterned structures [1,31,34]. The primary difficulty is producing a smooth cross-section through the cell and surface with minimal damage to the material along the split surface.

Common techniques to cross-section tissue samples include the use of a microtome or dual-beam techniques (focused ion beam (FIB) milling/scanning electron microscopy (SEM)); however, these two techniques often require infusing samples with media for mechanical support and protection during sample preparation. The infusion process may fill sub-surface voids beneath the cell or even damage existing fragile surface structures. Similarly, mechanical contact by a microtome blade may potentially damage material on the dissected surfaces. Dual-beam techniques have been used by the integrated circuit industry for defect inspection and circuit repair [35,36]. Researchers also use dual beam technique for sample cross-sectioning [37,38,39] and transmission electron microscopy sample preparation [36,40,41]. While this method has the advantage of being able to precisely target nanometer scale features, the technique is costly and requires significant sample preparation. Heavy milling ions, such as gallium, can also produce knock-on damage [42]. Milling by-products are often re-deposited nearby and can potentially fill sub-surface voids, which results in artifacts that cannot be detected when observing cells from the “top”. Finally, the milling process permanently removes material. Unlike with the use of microtome sectioning, there is no witness sample that remains for further inspection.

Without cross-sectioning the sample, others have looked at removing intact cells for inspection. For instance, Zhou et al. [21] peeled cells from the substrate and investigated the cell underbelly using atomic force microscopy. However, separating the cell from the surface will likely remove evidence of how the cell was attached to the surface. Non-destructive fluorescent confocal microscopy is another powerful method to inspect what is happening inside a cell. The technique has a unique advantage of being able to identify chemical components within the sample, which can then be used to digitally recreate a 3-D profile by z-stacking. However, the feature size and z-slice thickness are limited by the sensitivity of the photodetector, excitation wavelengths, as well as the availability and quality of the staining agents. The biggest drawbacks of confocal microscopy are that the technique cannot penetrate opaque materials, and fluorescent samples often have a short shelf life due to bleaching and other degradation mechanisms.

From the reports available in the literature, most techniques are prohibitively expensive, time consuming for large-scale sampling (observing more than 1 cell), and require specialized equipment. Hence, an inexpensive and fast cross-sectioning technique, with little sample preparation or mechanical contact/handling, is valuable to aid inspection of cells on surfaces. The driving hypothesis for this work was that novel insights could be obtained if there was a means to visually observe the underbelly of a mammalian cell (Vero) on textured tantalum surfaces. Therefore, the objective was to develop a simple inexpensive technique that allowed this observation and enhanced our knowledge of cell adhesion behavior on engineered tantalum surfaces. Tantalum is a bioactive material [43] commonly used in surgical implant and scaffold devices [44,45,46,47,48,49,50,51]. It has high mechanical strength and bioactivity [52,53]. Vero cells are commonly used in the virus production process in an anchorage-dependent process. Understanding how to control the cell morphology can potentially improve infection efficiency. Moussa et al [54] characterized the alignment behavior of Vero cells on tantalum parallel line comb structures and studied the morphology of thin pseudopodia and cellular materials located at the periphery of cells. However, they did not investigate the morphology in locations directly beneath the thick center portion of adherent cells—an area that is not easily accessible when working with opaque substrates. As a result, earlier studies in this area did not report on the influence of sub-cellular organelles, such as nucleolus, on the overall adhesion characteristics and cell morphology.

Herein, cells were cross-sectioned after fixation by using a three-point bend micro-cleaving technique and their underbelly morphology was characterized with high-resolution field-emission scanning electron microscopy (SEM). Field-emission SEM was used instead of fluorescence confocal microscopy because of the resolution power of the available SEM over that of the confocal microscope (~2 nm vs ~120 nm). While this cleaving technique does not have the precision targeting capability of dual-beam (FIB/SEM) techniques or the chemical identification capability of fluorescence confocal microscopy the cross-sectioning method is a complementary technique that has the advantage of being inexpensive, fast, and can be performed under ambient conditions. Depending on the cell concentration and the chip dimensions, it is possible to split thousands of cells at once. More importantly, no media infusion is needed. Results show that the underbelly is significantly more complex than what can be observed from top-down micrographs. More specifically, our micrographs show a large number of pseudopodia clusters jutted out from the bottom of the cell and attached to the bottom of trenches. This work has produced an invaluable technique to visualize nano-sized underbelly features that have never been reported before in literature.

## 2. Materials and Methods

### 2.1. Test Structure Fabrication

Nano- to micron-scale parallel line/trench comb structures were fabricated on 200 mm silicon substrates using high-precision advanced integrated circuit fabrication techniques and damascene integration methods [55,56,57]. These structures contained 0.18, 0.25, 0.5, 2, and 50 μm equal width trenches and lines. Samples were provided by Versum Materials, LLC (Tempe, AZ, USA). Photolithography and dry etching techniques [55,56,58] were used to transfer comb structure patterns to the silicon oxide coated substrate. Specimens were then coated with a thin layer of tantalum seed and copper. Excess copper was removed using a copper chemical-mechanical polishing technique [59,60]. Any remaining copper was chemically stripped with ~9.4 M diluted nitric acid ACS Plus (Fisherbrand®, Fisher Scientific International Inc., Pittsburgh, PA, USA) for ~45 min. Specimens were rinsed with deionized water and anhydrous ethanol prior to use. The final surface consisted of parallel trench structures that were conformably coated with ~20 nm of tantalum. The trench depth was ~700 nm. Representative SEM micrographs of the test structures are shown in Figure S1.

### 2.2. Cell Culture and Seeding

Cell culture procedures have been described elsewhere [2]. Briefly, Vero cells (ATCC, Manassas, VA, USA) were cultured in DMEM/F12 media (Corning, NY, USA), supplemented with 10% (v/v) Gibco™ fetal bovine serum (FBS, Thermo Fisher Scientific, Waltham, Massachusetts, USA) and 4-mM L-glutamine (Sigma-Aldrich, St. Louis, MO, USA). Patterned chips were sterilized using ethanol (70%) for 30 s followed by a rinse with Dulbecco’s phosphate-buffered saline (D-PBS) solution. After cell seeding with ~5 × 10^5^ cells/mL, specimens were incubation on the patterned structures in 5% CO_2_ atmosphere at 37 °C.

### 2.3. Cell Fixation and SEM

Adherent cells on the patterned structures were fixed with 4% methanol-free formaldehyde (Sigma-Aldrich, Oakville, Canada) for 1 h. After dehydrating the specimens with varying concentration of ethanol in water (50%, 75%, 95%, and 100% (v/v)) for at least 10 min each, samples were dried and stored in a nitrogen box. Specimens were inspected with a Zeiss 1550 field-emission scanning electron microscope (Carl Zeiss AG, Oberkochen, Germany) with the electron gun operating at 7 kV. None of the specimens were coated with conducting materials prior to imaging.

### 2.4. Specimen Cross-Sectioning

**Figure 1:**
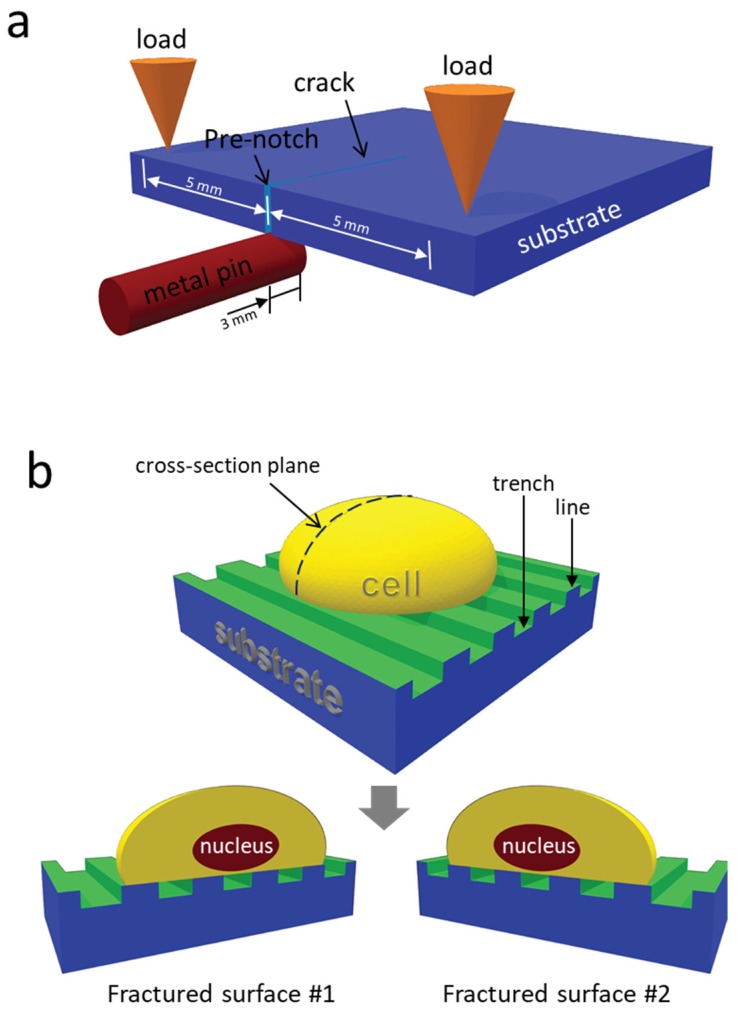
(**a**) Schematic drawing of the three-point bend micro-cleaving. (**b**) Cell dissection obtained using three-point bend technique. Substrate is covered with ~20 nm tantalum film (green).

Dried specimens were cross-sectioned using a three-point bend cleaving technique. No infusion media was needed prior to cleaving. The location and orientation of the three contact points are illustrated in Figure 1a. A pre-notch approximately 0.5 mm long was created by pressing and scratching a diamond scribe tip on the edge of the substrate. A thin metal pin (~1 mm diameter) was placed beneath the notch as the central pivot point and extended ~3 mm under the substrate. Two external loads, applied with fingers at ~5 mm from the center pin, were pressed on the sample edge to complete the three-point bend configuration. When the manually applied load was larger than the critical fracture load limit, a single crack emanated from the notch and propagated across the entire device. A schematic drawing of an adherent cell on a parallel line/trench comb structure before and after cleaving is illustrated in Figure 1b. Both fractured surfaces created by this cleaving technique were inspected using high-resolution field-emission SEM.

### 2.5. Cell Fixation, Staining, and Confocal Fluorescence Microscopy

Detail procedure for cell fixation, staining and fluorescence confocal microscopy has been described in detail elsewhere [3,54]. Briefly, cells were fixed with 4% methanol-free formaldehyde (Sigma-Aldrich, Oakville, Canada) after incubation. Specimens were permeabilized with 0.1% Triton-X 100 (Sigma-Aldrich, Oakville, Canada) prior to staining cells with 4′,6-diamidino-2-phenylindole (DAPI) (Life Technologies, Waltham, MA, USA) and Phalloidin-iFluor 633 Reagent-CytoPainter (ab176758 Abcam, Abcam Inc., Cambridge, MA, USA). Dulbecco’s phosphate-buffered saline solution was used to rinse samples in between each step. Prolong Gold antifade reagent (Life Technologies, USA) was added to specimens in the final step to extend the chemical stain lifetime. A Leica TCS SP5 confocal fluorescence microscope (Leica, Wetzlar, Germany) operating under refection configuration was used to inspect the stained cells.

## 3. Results and Discussions

### 3.1. Fluorescence Confocal Microscopy

**Figure 2:**
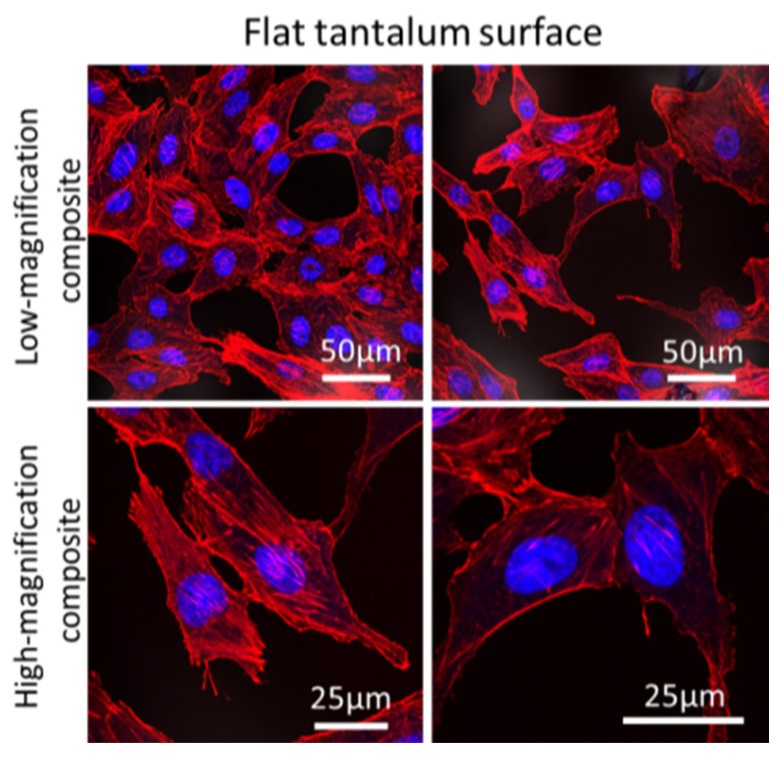
Fluorescence confocal micrographs show cell nuclei (blue) and actin micro-filaments (red) randomly oriented on blanket tantalum surfaces.

**Figure 3:**
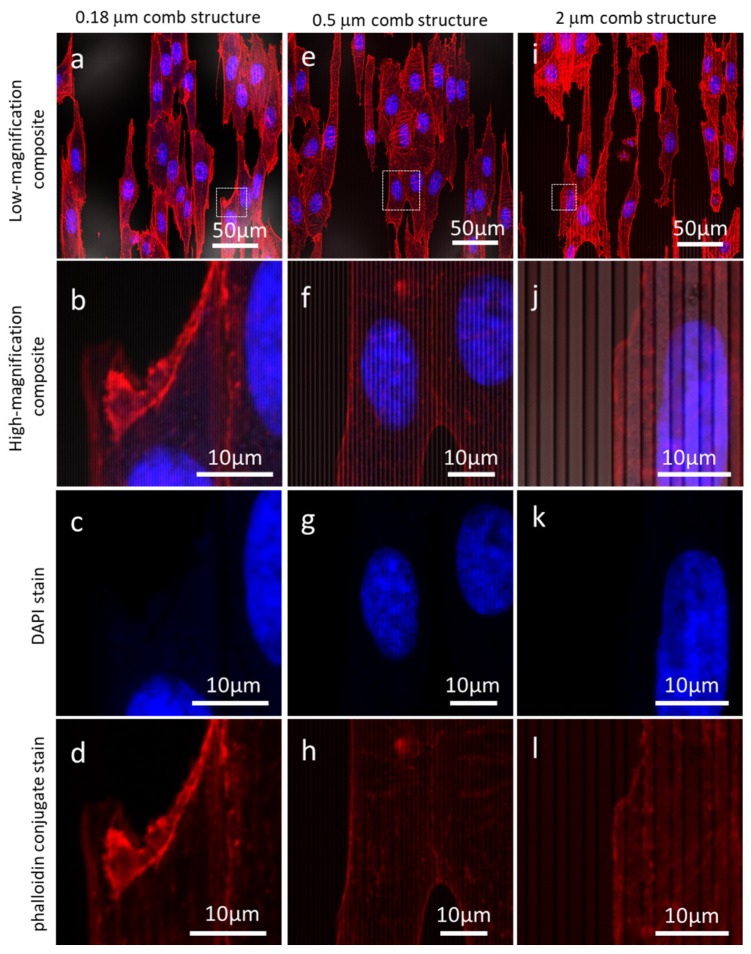
Low- and high-magnification fluorescence confocal micrographs of cells adhered to 0.18 μm (**a**–**d**), 0.5 μm (**e**–**h**), and 2 μm comb structures (**i**–**l**). The nuclei (blue), actin microfilaments (red) aligned to the pattern axes. Cells were incubated for 24 h with a concentration of ~5 × 10^5^ cells/mL.

Since the tantalum coated silicon substrata is opaque and its surface is reflective to light, common transmission microscopy methods cannot be applied to these specimens. Therefore, the morphology of the Vero cells was characterized using reflection fluorescence confocal microscopy. Micrographs of cells adhered to the flat blanket tantalum surface and parallel line comb structures are shown in Figure 2 and Figure 3, respectively. The cell nuclei (blue) and actin micro-filaments (red) were randomly oriented on the flat tantalum surfaces (Figure 2), while cells on the 0.18 μm, 0.5 μm, and 2 μm comb structures exhibited preferential alignment to the line axes. The parallel line structures (light gray) were included in the composite image. Detailed quantitative analysis of this surface topography induced cell alignment behavior has been discussed elsewhere [54].

Close inspection of the high-magnification confocal micrographs showed that fluorescence signals from DAPI (Figure 3c,g,k)) and phalloidin stains (Figure 3d,h,l)) were generally more intense on the lines but weaker at the trench and sidewall locations. It is unclear if these localized intensity patterns represent genuine concentration gradients of the proteins or if they are artifacts induced by the reflection and diffraction of fluorescence signal on the tantalum coated 3D surface. For example, faint parallel line structures were observed on the phalloidin micrographs (Figure 3h,l, and Figure S2) while greater fluorescence intensity was observed on lines compared to the trenches and the sidewalls. In contrast, no background feature was observed on the flat blanket tantalum substrate shown in Figure 2. Additional confocal micrographs with line patterned intensity and background of line structures are shown in Figure S2.

High-magnification reflection confocal micrographs displayed in Figure 3 and Figure S2 also show the difficulties in resolving individual fluorescence feature with effective diameters smaller than ~100 nm. It is particularly problematic to isolate signals from the trenches or sidewall regions where the background intensity is smaller than on the lines. Hence, a method was needed to cross-section the specimens and directly inspect the cellular structures in the trenches to avoid background artifacts that do not interfere with the results.

### 3.2. Impact of Cleaving on Sample Integrity

**Figure 4:**
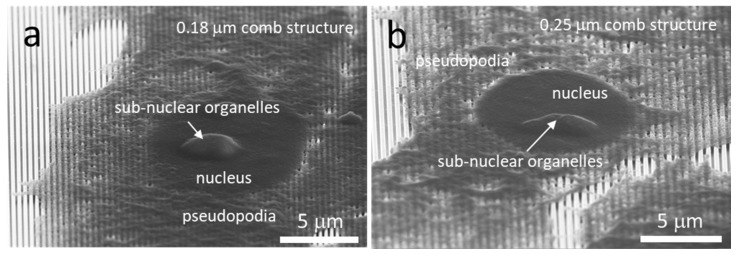
Typical 70° tilted low-magnification SEM micrographs of cells on comb structures with trench widths of (**a**) 0.18 μm and (**b**) 0.25 μm. Note that cellular material within the trenches is not visible. A cross-sectioning technique is needed to visualize the underbelly of the cell.

Representative 70 degree tilted scanning electron micrographs (SEM) of cells on the 0.18 and 0.25 μm comb structures are shown in Figure 4a,b respectively. These cells were incubated on the substrates for 24 h. Micrographs show the periphery of the cell was thin and appeared to float on the engineered features. In contrast, the cells are thicker near the nucleus which contains large dome-shaped sub-nuclear organelle, possibly the nucleolus. The thicker portions of the cell mask how the cell is actually conforming to the surface beneath. Cross-sectioning of these specimens is necessary to understand the cell-substrate interface adhesion, especially around the location of the nucleus.

**Figure 5:**
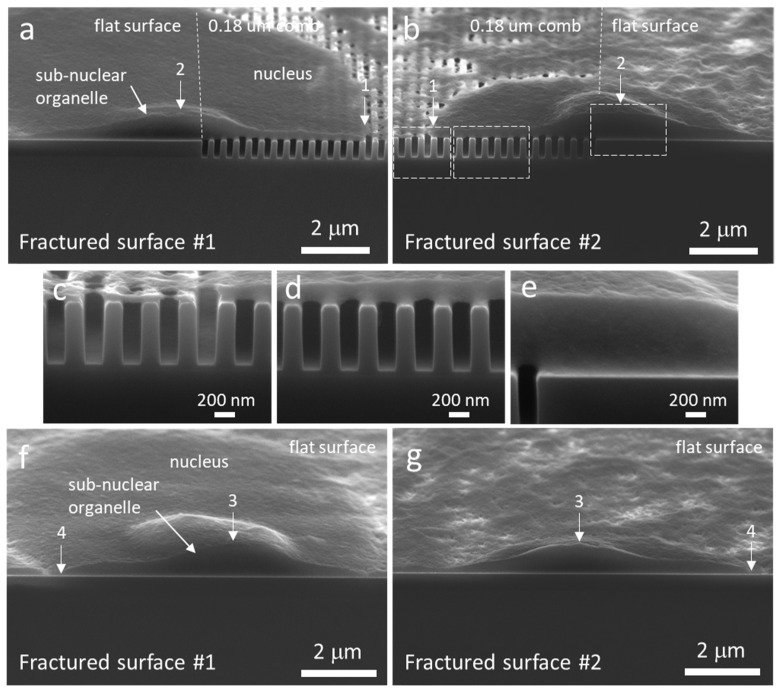
Tilted SEM micrographs of two cross-sectioned cells. (**a**–**b**) Cell adhered on two types of surface (flat and 0.18 μm trench pattern). (**c**–**e**) High magnification images of the fractured surface in (**b**). (**f**–**g**) Cell adhered solely on a flat surface. Both sides of the cleaved surfaces are displayed in the figure (left and right columns of images). Landmarks “1–“4” are used to match the topographic features on the two fractured surfaces. In both cells, a micron size sub-nuclear organelle was cross-sectioned.

To evaluate the impact of the 3-point bend cleaving process on material adjacent to the fractured surface, cross-sectioned specimens were inspected by using SEM (Figure 5). Typical low- and high-magnification micrographs of a cell located at the boundary between the 0.18 μm comb structure and the flat surface is shown in Figure 5a–e, while a cell that adhered entirely on the flat surface is displayed in Figure 5f,g. The dome-shaped sub-nuclear organelle was successfully cross-sectioned in both cells. Images labeled with fractured surfaces (#1 and #2) correspond to the two separated surfaces of each specimen. Landmarks “1”–“4” labeled on the images are used to aid viewers to match the topographic features on both sides of the fractured surfaces. There is no evidence observable gross damage of the surfaces. Figure 5a revealed the cell extended ~4.6 μm into the 0.18 μm comb structure area. These micrographs confirmed that cellular materials did not descend into these open trenches. Results also demonstrated that the micro-cleaving technique was used to cross-section adherent cells and did not produce observable damage to material adjacent to the fractured surfaces. Additional high-magnification micrographs of cells on comb structures with various trench widths are provided in Figure S3a–e.

### 3.3. Cell Cross-Sectional Morphology on the Comb Structures

**Figure 6:**
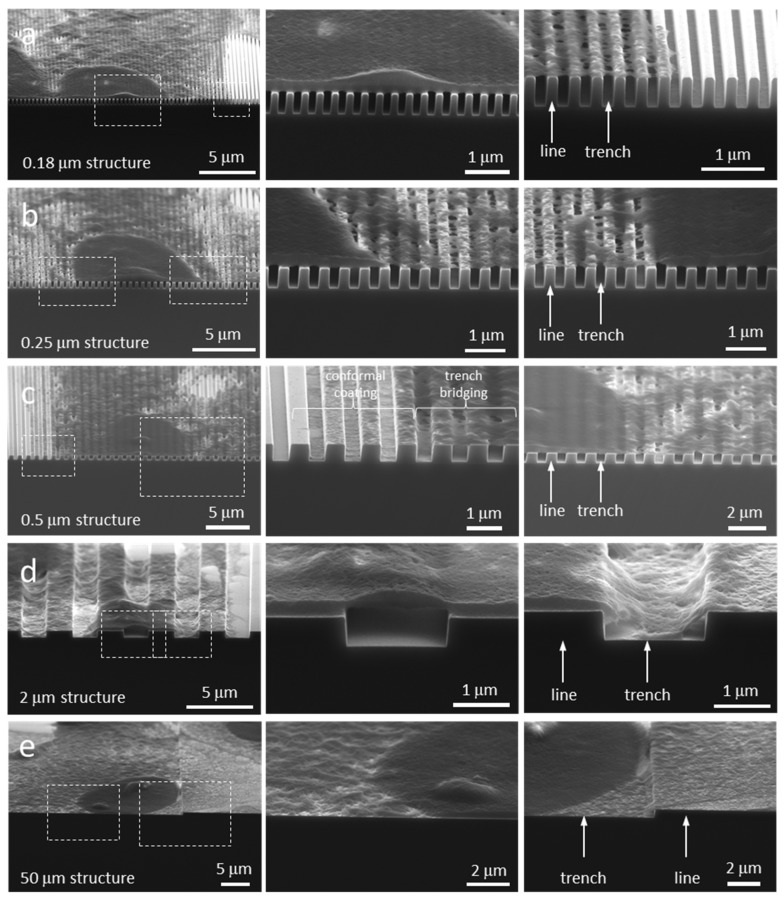
Typical SEM micrographs of cross-sectioned cells on the comb structures with trench widths of (**a**) 0.18, (**b**) 0.25, (**c**) 0.5, (**d**) 2, and (**e**) 50 μm.

**Figure 7:**
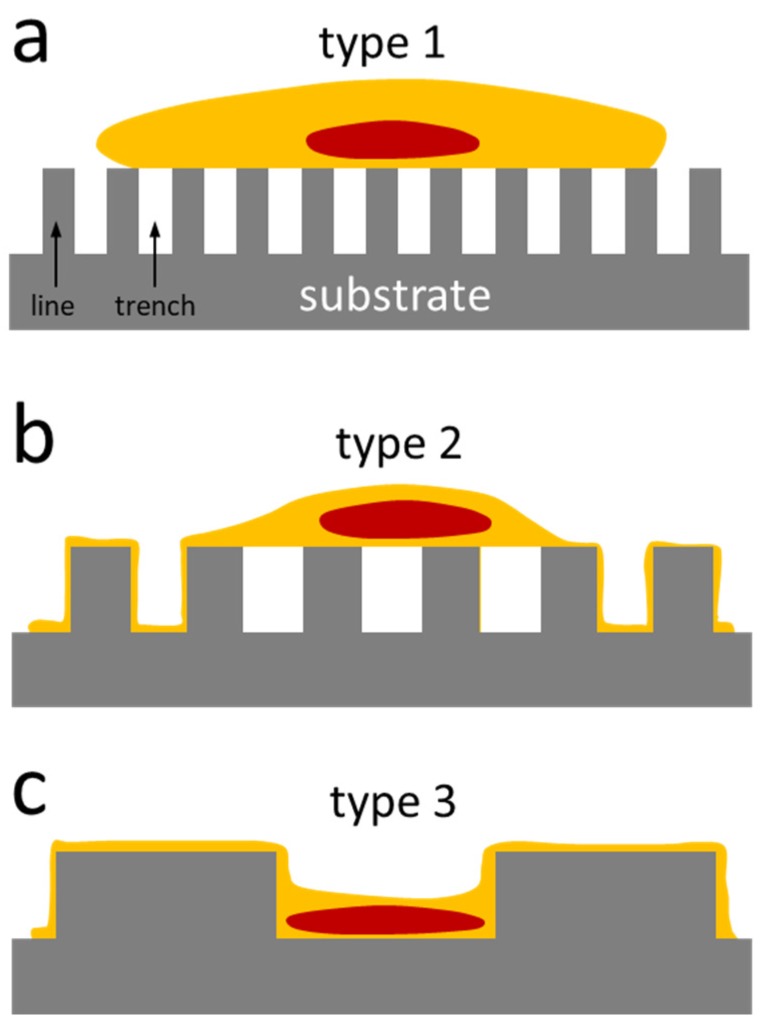
Schematic drawings of three different types of cell cross-sectional morphology observed with increasing line and trench widths. (**a**) Cell “floated” on top of the comb structure; (**b**) pseudopodia located at the edge of cells descended into the trenches; (**c**) entire cell including the nucleus formed a conformal coating on the comb structure surface.

Examples of cross-sectioned cells on 0.18, 0.25, 0.5, 2, and 50 μm comb structures are displayed in Figure 6a–e respectively. The number of cells inspected on each comb structure and their corresponding morphology type are summarized in Table 1. Micrographs show cells “floating” on top of the 0.18 and 0.25 μm parallel trench structures. There was no evidence that any cellular components descended into these trenches. This type of cell cross-sectional morphology, denoted as Type 1, is schematically illustrated in Figure 7a. As the trench widths increased, pseudopodia were observed to fill the trench openings (Figure 6c,d). This morphology was categorized as Type 2 (schematically illustrated in Figure 7b). Pseudopodia located at the edge of cells follow the 0.5 μm comb structures surface topography tightly while the remaining part of cell float on the trenches (Figure 6c). In contrast, all but the center portion of nucleus descended into the trench when cells adhered on the 2 μm comb structure (see Figure 6d). Cells on comb structures with wide trenches exhibited what has been denoted as Type 3 morphology (Figure 7c), where the entire cell including the nucleus formed a conformal coating on the surface (Figure 6e). Additional examples of cross-sectioned cells on various comb structures are shown in Figures S4–S8 respectively. These images demonstrate the reproducibility and reliability of this cross-sectioning technique for observing cells on our devices.

The micrographs and schematic drawings shown in Figure 6 and Figure 7 indicated that the cell cross-sectional morphology is complex and difficult to generalize. Results clearly demonstrated that multiple cross-sectional morphologies co-existed within a single cell and the proportion of each depended on the surface features. Nuclei were less likely to descend into the trenches, a result supported by the fact that the cell nuclei are thicker and stiffer than other cellular components [31,32]. Our findings suggest a deficiency of existing cell adhesion models [20,22], which primarily focus on the molecular mechanisms of adhesion at the plasma membranes and do not account for the physical contributions by sub-cellular organelles. Results presented here will allow researchers to develop a more comprehensive and accurate model for cell adhesion and tissue engineering.

### 3.4. Mechanisms of Nucleus Migration into Trenches

**Figure 8:**
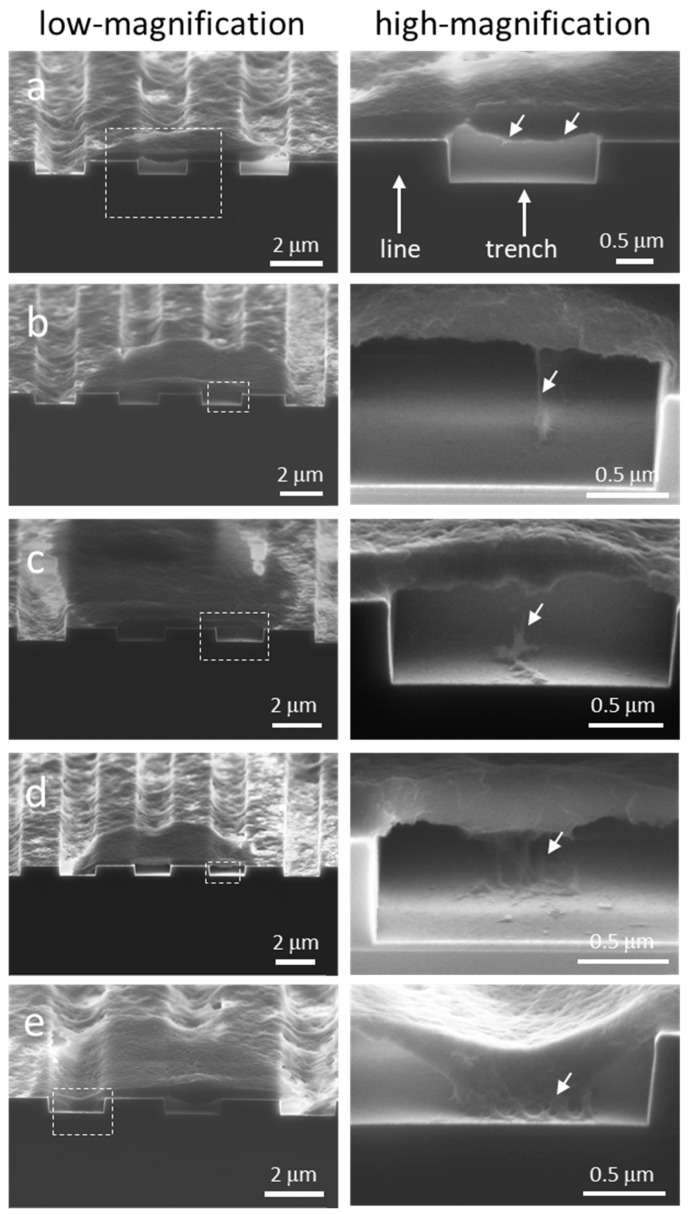
Micrographs of dissected cells show different amounts of cellular material descending into the 2 μm trenches. The size of filament cluster or fibrous structures that connect the cell underbelly to the substrate increased as more materials sunk into the trench. (**a**) Short filaments protruding out of the cell underbelly but that did not attach to any surface; (**b**-**c**) single-strand filaments from the cell underbelly attached to the bottom of trenches; (**d**) a cluster of filaments connected the cell underbelly and the bottom of trenches; (**e**) a large network of fibrous structures is formed between the cell and the trench bottom surfaces.

While micrographs in Figure 6 show cellular materials descended into the trenches, it is unclear the mechanisms of this process. We are particularly interested in the nucleus because it is one of the stiffest organelles in the cell and it is likely to be the least flexible part of the cell to fit into surface openings. To understand this mechanism, cells adhered on the 2 μm comb device were cross-sectioned and inspected using high-magnification SEM. The structure had lines and trenches with equal widths of 2 μm. Micrographs of dissected cells with different amounts of cellular material descending into the trenches are shown in Figure 8a–e, respectively. These images were focused on the cell underbelly, directly beneath the thick nucleus. Figure 8a shows an empty trench with two short filaments, highlighted with arrows, protruding out of the cell underbelly but that did not attach to any surface. Cells with longer filaments extended from the cell underbelly and attached to the bottom surface of the trench (Figure 8b,c). These short filaments had effective diameters on the order of ~50 nm, which agree with the minimal pseudopodia dimension to form stable focal adhesion [4]. Other than the filaments, there is no other cellular material that descended into these two trenches. A micrograph of a cross-sectioned cell nucleus that was partially sunk into the trench is displayed in Figure 8d. The images show a cluster of vertically aligned filaments connecting the underbelly of the cell to the bottom surface of the trench. The effective diameter of each filament was ~ 50 nm, similar to those single-strand filaments observed in Figure 8b,c. An example of a nucleus that is sunk deep into the trench, descending to within 100 nm from the bottom surface of the trench, is shown in Figure 8e. In this case, there is a large network of fibrous structures connected the bottom of this cell depression and the substrate surface. Micrographs displayed in Figure 8 show that as more cellular material descended into the trench, the size of the filament clusters or fibrous structures that connected the cell underbelly to the substrate also increased. More interestingly, all of the anchor points of these structures were located near the center of the trenches. None were connected to the sidewalls even though all trench surfaces were coated with tantalum. Even after 24 h of incubation, nano- to micron-scale cavities remained near the sidewalls as shown in Figure 8e. It is unclear if these cavities eventually became filled with cellular materials. This is the first demonstration that the sidewalls had little to no contribution to the migration of the nucleus into the trenches.

The underbelly morphology presented in Figure 8 appears to be different from those reported by Zhou et al. [21], who studied the adhesion behaviors of HeLa cells on parallel trench comb structures. Their atomic force microscopy (AFM) surface profiles of cell underbelly suggested that the cell descends into the trench opening without the presence of nanometer scale filaments or fibrous structures. The difference is likely related to the sample preparation technique. Their cells were peeled off from the patterned substrate and then imaged by AFM. It is unclear the amount of damage that may have been introduced during this separation procedure. Fragile elements such as the nanometer scale cellular features shown in Figure 8 could easily be damaged or deformed during the peeling process.

### 3.5. Possible Impact of Sub-Nuclear Organelles on Cell Cross-Sectional Morphology

**Figure 9:**
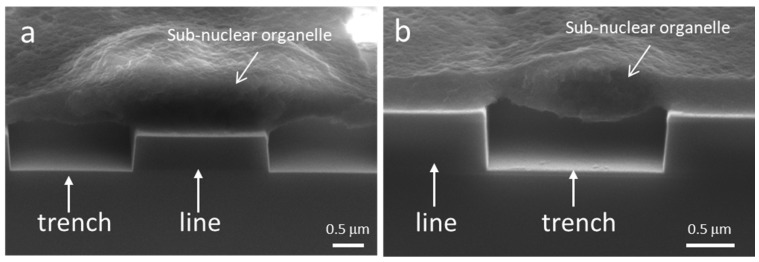
Micrographs show cell adhered to the 2 μm comb structures. (**a**) The sub-nuclear organelle resting on the line is shown in (**a**) while the organelle sat in the trench is shown in (**b**).

Organelles can potentially influence cell morphology because of their mechanical properties. An example to illustrate the potential impact of an organelle is shown in Figure 9. The micrographs show two cross-sectioned cell nuclei containing dome-shaped sub-nuclear organelles. One had a thickness of ~870 nm and adhered on a line structure (Figure 9a). The other organelle had a thickness of ~610 nm and was part of a bridge formed across a trench (Figure 9b). These organelles were at least ~1.8 times thicker than the surrounding nuclear material (~320–340 nm). When a thick organelle adheres on a line structure, the organelle contributes to increasing the effective thickness and stiffness of adjacent trench bridge structure. Hence, when an organelle falls on a line structure, more force may be needed to cause cellular material to descend into the trenches. In contrast, when large organelles become part of a trench bridge as shown in Figure 9b the effective vertical distance between the cell underbelly and trench bottom surface is reduced significantly. This may facilitate cell adhesion as shorter filaments are required to connect the cell and the trench bottom surface. Another possible impact with an organelle settled into a trench, is that it may hinder the cell movement in directions perpendicular to the line axes. This could be because the organelles change the local cell surface topography, which can increase the friction between the cell and the patterned surface.

In summary, this work demonstrated that a 3-point bend cleaving method can be a useful tool to investigate the cell underbelly morphology. There are numerous research questions that can be explored with this technique. For example, the capability to dissect the nucleus and visualize its content will allow research into viral infection processes by allowing the inspection of virus distribution, populations, and proliferation rates within the nucleus. Another possible application is the investigation of non-viral transfection efficiencies on surfaces with different pillar densities. While transfection efficiencies on different surfaces have been reported [61], none have been able to show how the underbelly plays a role in aiding the process. In addition to work on eukaryotic cells, a cross-sectioning technique, such as the one presented here, is valuable for studying bacterial cells and biofilms on nanostructured surfaces. For example, bacteria, such as *Staphylococcus aureus*, are a significant pathogen responsible for nosocomial infections and studies investigating how they interact migrate, and transport across surfaces will have a huge impact on the design and generation of materials to be used in hospital settings. While this work has shown results for cells on silicon substrates, the current sample preparation technique can be applied to other materials, including glass and ceramics. Polymer and metal surfaces can also be cleaved by this method so long as the temperature of the material is below its ductile-to-brittle transition temperature.

## 4. Conclusions

Mammalian cells and their nuclei were successfully dissected using a micro-cleaving technique and inspected using high-resolution SEM. Results showed that cells “floated” on top of the dense comb structures with trench widths smaller than 0.5 μm. Increasing amounts of cellular material descended into the opening as the widths of the trenches became wider. The morphology of cross-sectioned cells on 0.5 and 2 μm comb structures were not uniform. Pseudopodia located at the cell perimeter formed a conformal coating to the surface topography while regions near the nucleus floated on the comb structure. For the first time, SEM micrographs have been obtained that reveal cellular material immediately beneath the nucleus descending into the trench opening. This process involved nano-meter scale filaments jutting out of the cell underbelly and connecting to the mid-point of the surface at the bottom of the trench. Furthermore, the micrographs revealed that the process did not involve adhesion with the sidewalls. The size of the filament clusters or fibrous structures that connected the cell underbelly to the substrate increased as more materials sunk into the trench. These new findings will help researchers develop a more comprehensive model of cell adhesion behavior. In our hands, the capability of this technique to dissect nuclei and its internal organelles may provide an invaluable tool to study how viruses proliferate and form within a cell.

## Figures and Tables

**Table 1:** Number of cells inspected in each comb structure and the corresponding type of morphology.

Comb Structure Line and Trench Width (μm)	Number of Cells Inspected	Morphology Type
0.18	15	1
0.25	11	1
0.5	18	2
2.0	18	2
50	7	3

## Supplementary Materials

The following are available online at http://www.mdpi.com/1996-1944/12/1/114/s1, Figure S1: Typical SEM micrographs of comb structures with line and trench widths of (a) 1 μm and (b) 0.5 μm. Scale bars represent 1 μm, Figure S2a: Low- and high-magnification fluorescence confocal micrographs of cells on 2 μm comb structures, Figure S2b: Low- and high-magnification fluorescence confocal micrographs of cells on 0.5 μm comb structures, Figure S2c: Low- and high-magnification fluorescence confocal micrographs of cells on 0.18 μm comb structures, Figure S2d: Low- and high-magnification fluorescence confocal micrographs of cells on 0.18 μm comb structures, Figure S3a: Low- and high-magnification SEM micrographs of comb structures with line and trench widths of 0.18 μm, Figure S3b: Low- and high-magnification SEM micrographs of comb structures with line and trench widths of 0.18 μmm, Figure S3c: Low- and high-magnification SEM micrographs of comb structures with line and trench widths of 0.25 μm, Figure S3d: Low- and high-magnification SEM micrographs of comb structures with line and trench widths of 0.25 μm, Figure S3e: Low- and high-magnification SEM micrographs of comb structures with line and trench widths of 50 μm, Figure S4: Pseudopodia morphology of cells on the 0.18 μm comb structure. High-magnification images show the nanometer scale pseudopodia filaments bridged across the trench structures. More importantly, micrographs show the cleaving process did not produce observable damages to the patterned line structures and the cells. Nanometer scale filaments can be seen bridging the trenches adjacent to the fractured surfaces, Figure S5: Tilted SEM micrographs of three cross-sectioned cells (a–c). All cells are adhered to the 0.18 μm patterned structure. Landmarks “1”–“6” are visual aids to match the surface features, Figure S6: Tilted SEM micrographs of two cross-sectioned cells (a) and (b). (a) Cell is adhered on two surface structures–flat surface and 0.18 μm patterned structure. (b) Entire cell is on the 0.18 μm comb structure. Both fractured surfaces are displayed (left and right columns of images). Landmarks “1”–“6” are used to aid viewers to match the surface features. Dash lines in (a) mark the boundaries between the two patterned surfaces, Figure S7: Tilted SEM micrographs of two cross-sectioned cells (a) and (b) on the 0.5 μm patterned structure. Landmarks “1”–“4” are used to aid matching the surface features, Figure S8: Tilted SEM micrographs of a cross-sectional cell adhered on the 2 μm trench patterned structure. Landmarks “1”–“3” are used to aid matching the surface features. Note the center portion of this nucleus bridges across the trench while the rest of the cell conformal coat the surface topography.
