# Supplementary material for: What’s Happening on the Other Side? Revealing Nano-Meter Scale Features of Mammalian Cells on Engineered Textured Tantalum Surfaces
Source: Materials (Basel). 2018 Dec 31;12(1):114. doi: 10.3390/ma12010114 (PMC6337376; doi:10.3390/ma12010114)
Supplement: Supplementary file 1 [file materials-12-00114-s001.pdf]

materials

MDPI

Supplementary Materials

What's Happening on the Other Side? Revealing Nano-Meter Scale Features of Mammalian Cells on Engineered Textured Tantalum Surfaces

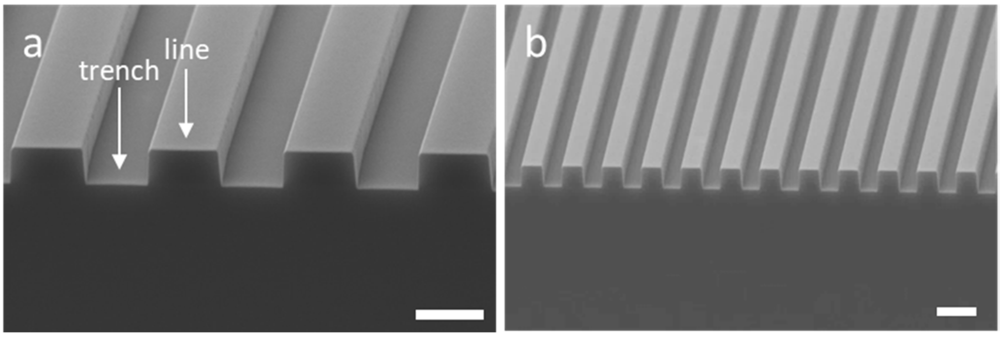

Figure S1. Typical SEM micrographs of comb structures with line and trench widths of (**a**) 1 μm and (**b**) 0.5 μm. Scale bars represent 1 μm.

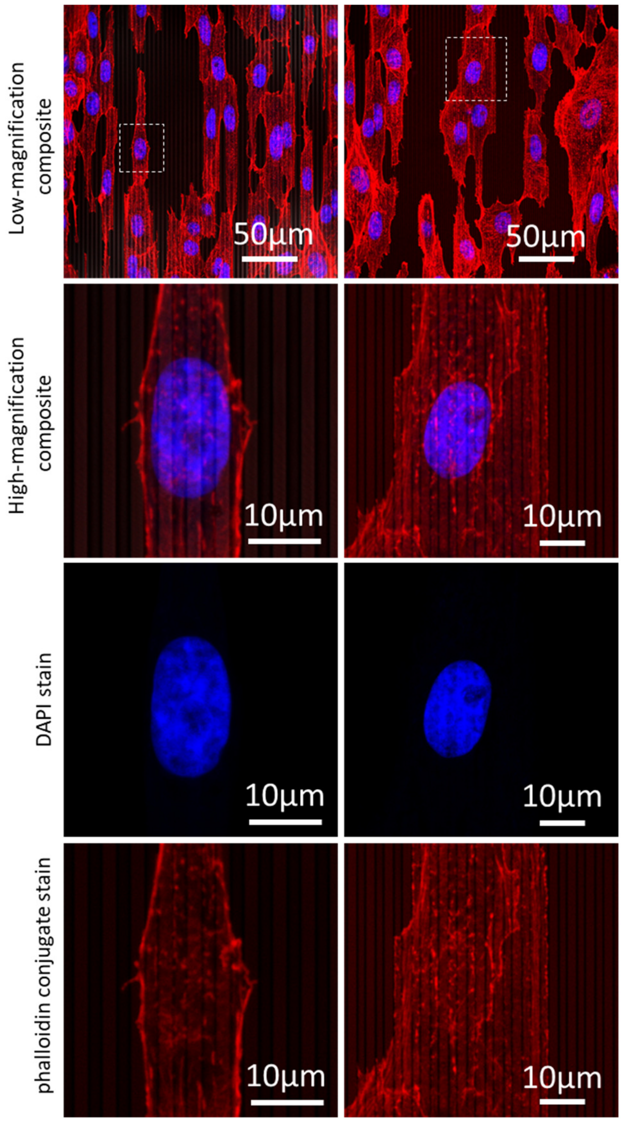

Figure S2a. Low- and high-magnification fluorescence confocal micrographs of cells on 2 µm comb structures.

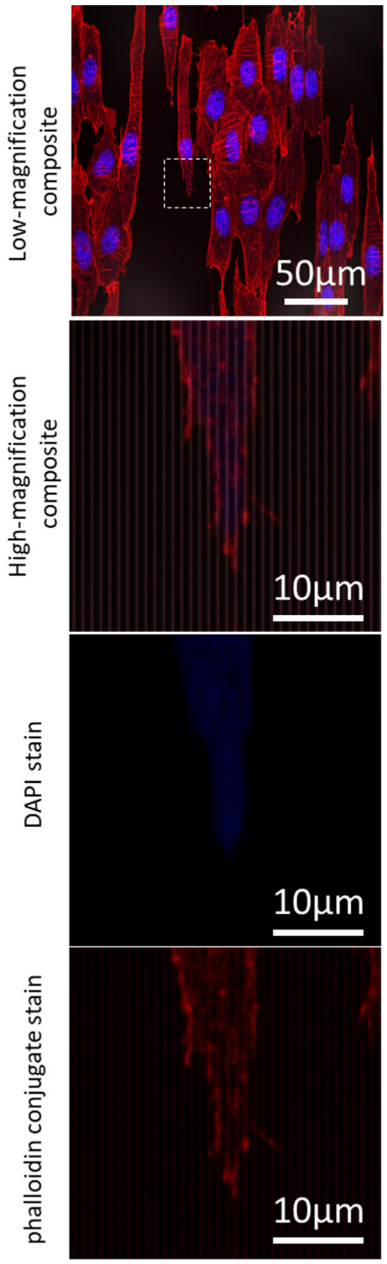

Figure S2b. Low- and high-magnification fluorescence confocal micrographs of cells on 0.5 µm comb structures.

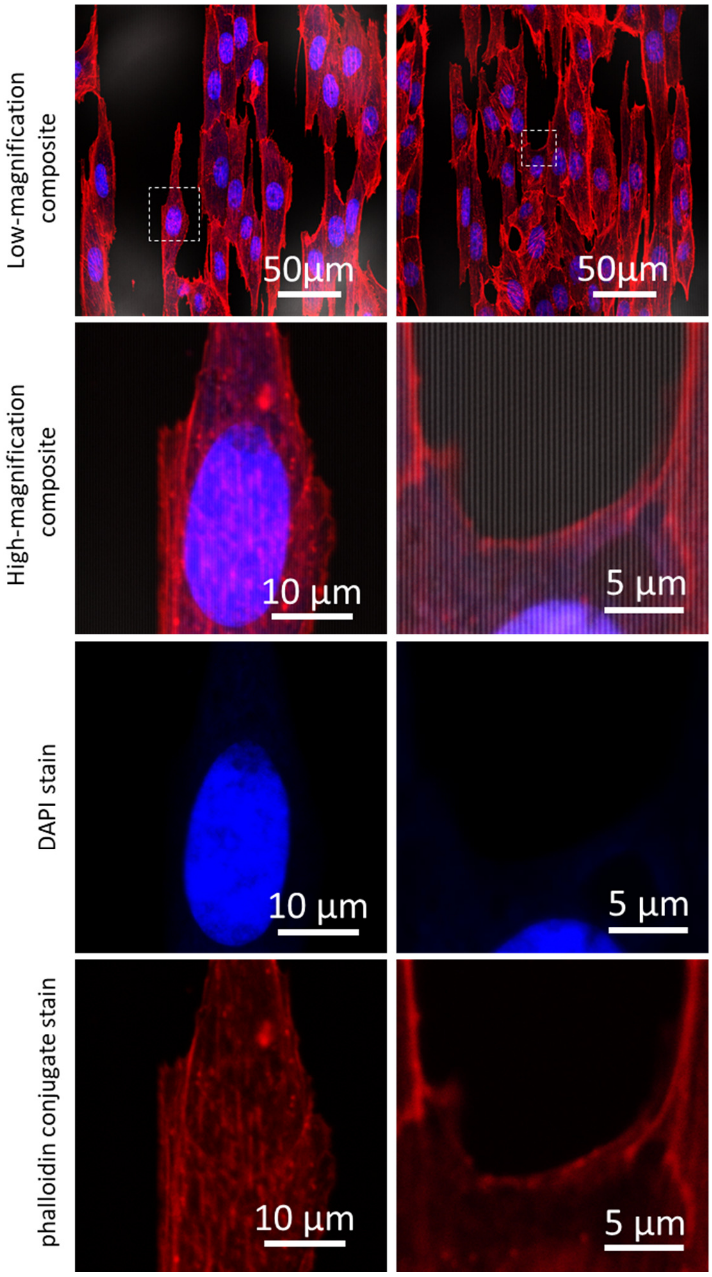

Figure S2c. Low- and high-magnification fluorescence confocal micrographs of cells on 0.18 µm comb structures.

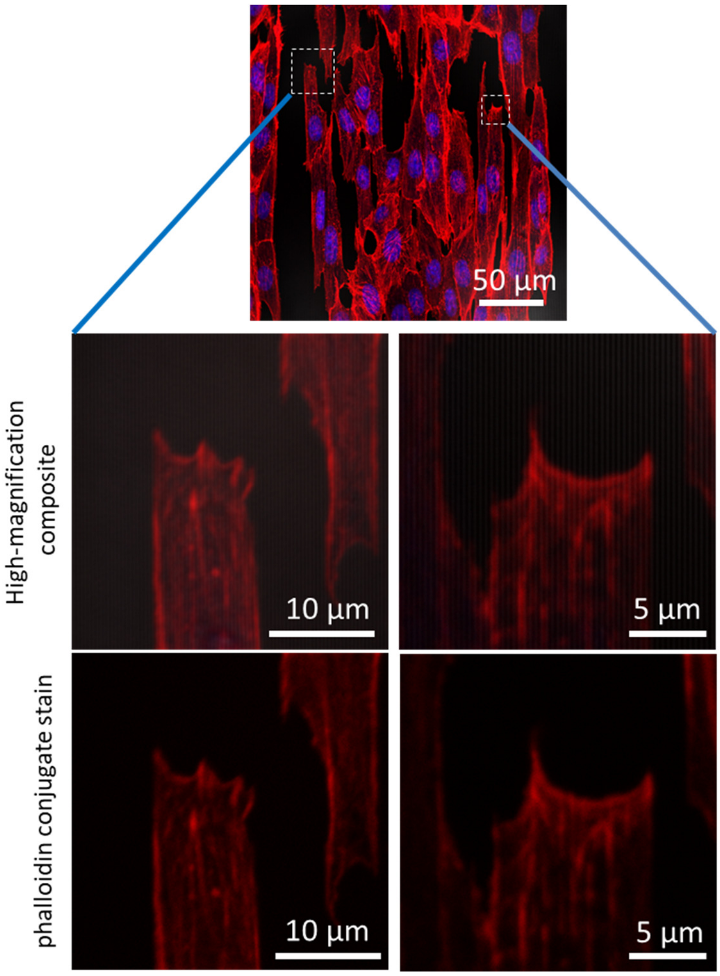

Figure S2d. Low- and high-magnification fluorescence confocal micrographs of cells on 0.18 µm comb structures.

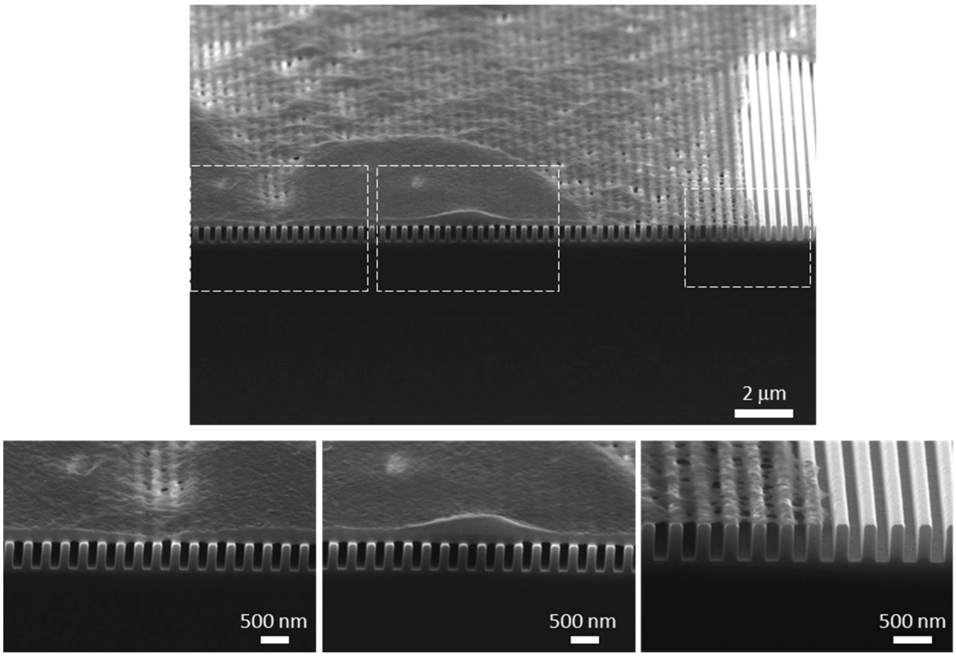

Figure S3a. Low- and high-magnification SEM micrographs of comb structures with line and trench widths of 0.18 µm.

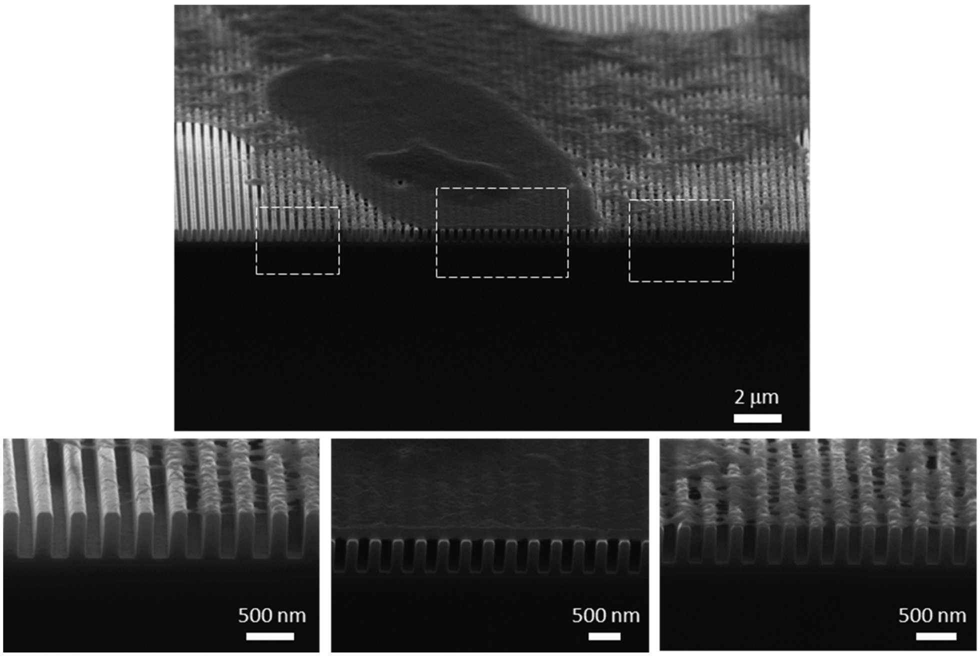

Figure S3b. Low- and high-magnification SEM micrographs of comb structures with line and trench widths of 0.18 µm.

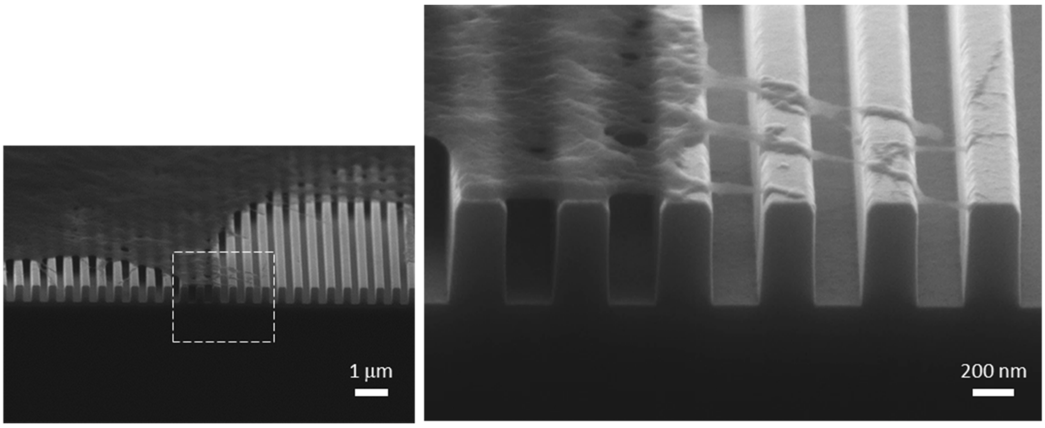

Figure S3c. Low- and high-magnification SEM micrographs of comb structures with line and trench widths of 0.25 µm.

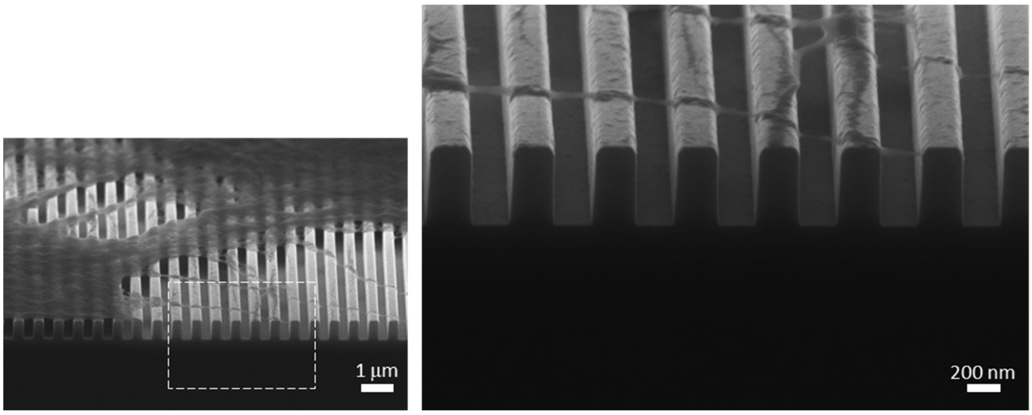

Figure S3d. Low- and high-magnification SEM micrographs of comb structures with line and trench widths of 0.25 µm.

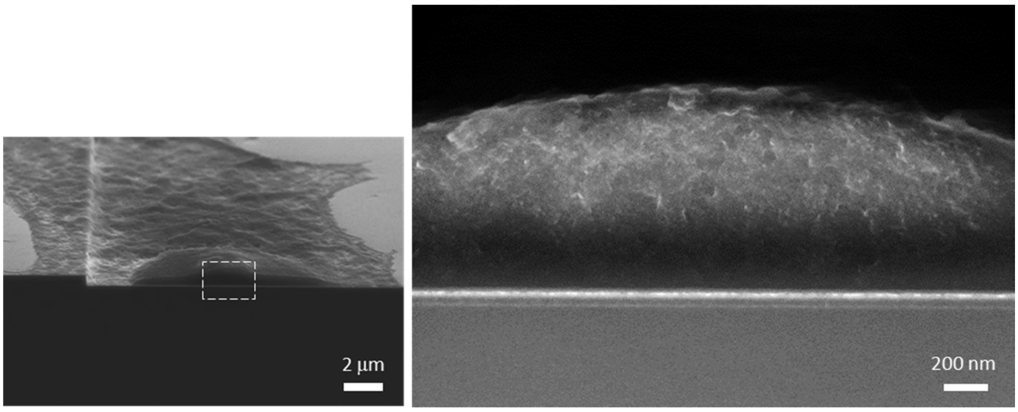

Figure S3e. Low- and high-magnification SEM micrographs of comb structures with line and trench widths of 50 µm.

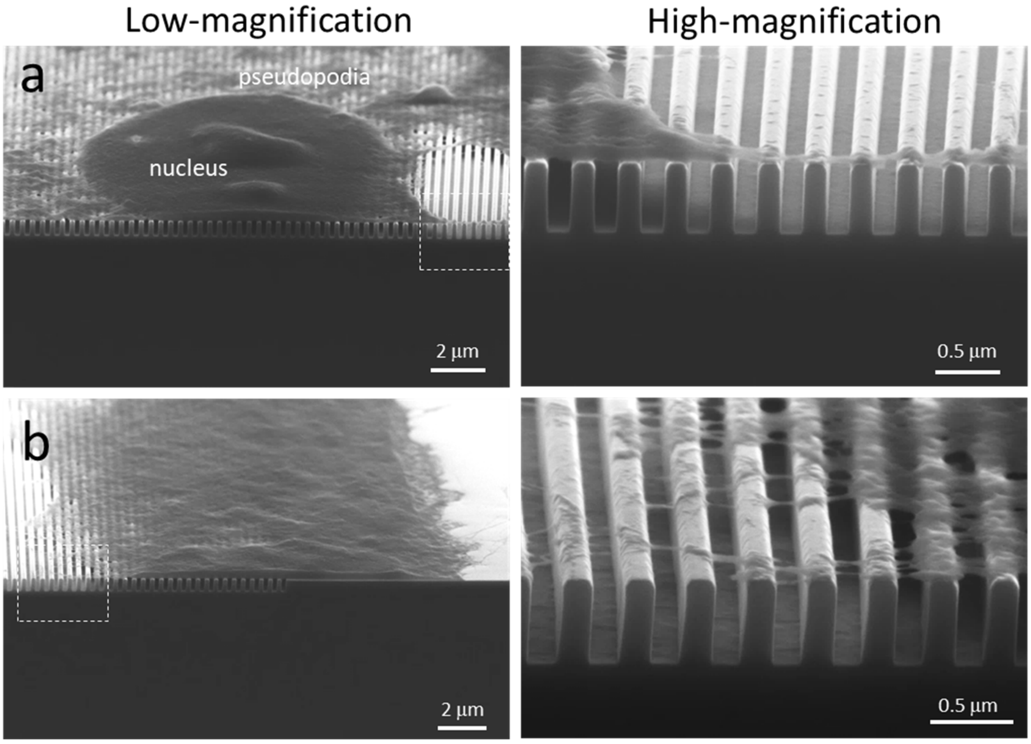

Figure S4. Pseudopodia morphology of cells on the 0.18 µm comb structure. High-magnification images show the nanometer scale pseudopodia filaments bridged across the trench structures. More importantly, micrographs show the cleaving process did not produce observable damages to the patterned line structures and the cells. Nanometer scale filaments can be seen bridging the trenches adjacent to the fractured surfaces.

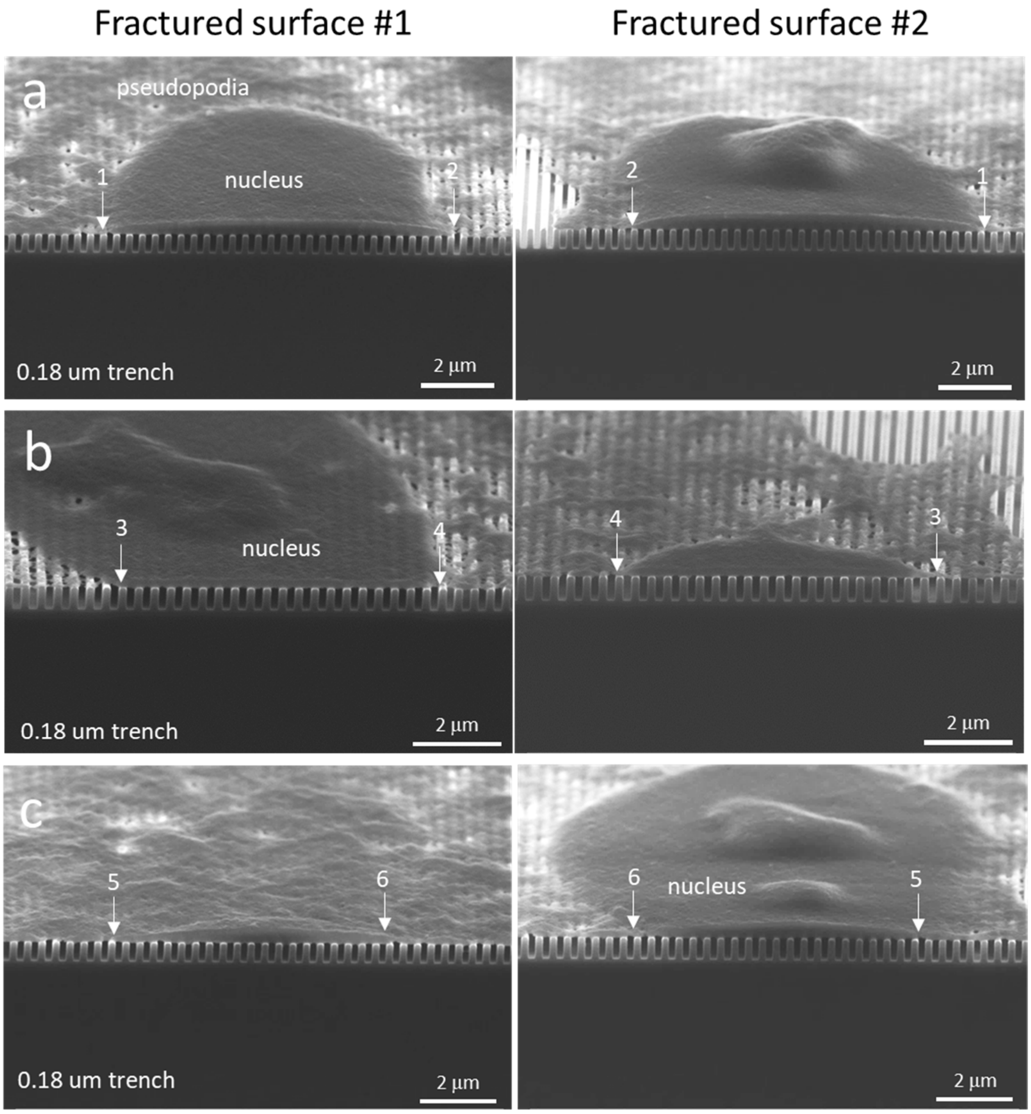

Figure S5. Tilted SEM micrographs of three cross-sectioned cells (**a**–**c**). All cells are adhered to the 0.18 μm patterned structure. Landmarks "1"–"6" are visual aids to match the surface features.

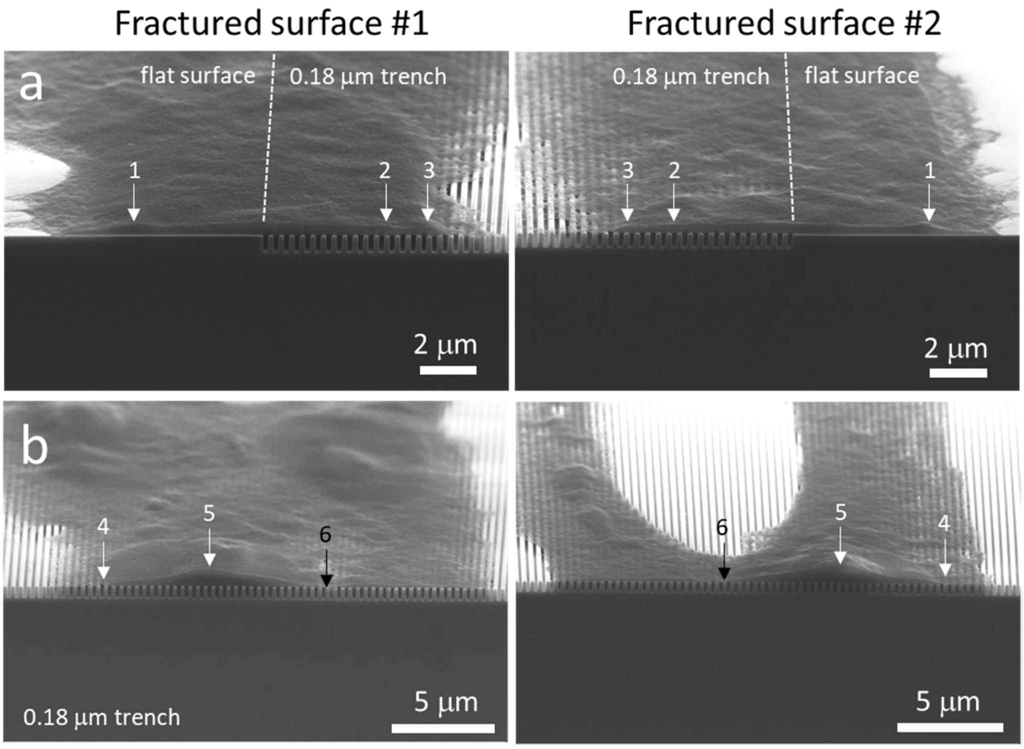

Figure S6. Tilted SEM micrographs of two cross-sectioned cells (**a**) and (**b**). (**a**) Cell is adhered on two surface structures–flat surface and 0.18 µm patterned structure. (**b**) Entire cell is on the 0.18 µm comb structure. Both fractured surfaces are displayed (left and right columns of images). Landmarks "1"–"6" are used to aid viewers to match the surface features. Dash lines in (**a**) mark the boundaries between the two patterned surfaces.

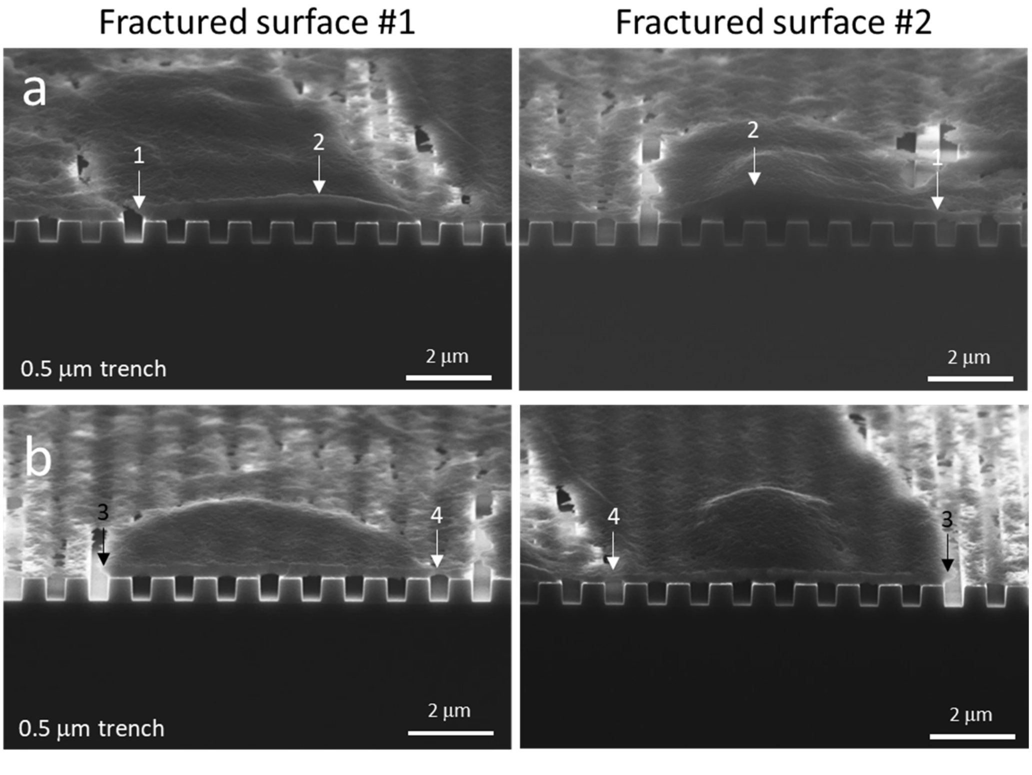

Figure S7. Tilted SEM micrographs of two cross-sectioned cells (**a**) and (**b**) on the 0.5 µm patterned structure. Landmarks "1"–"4" are used to aid matching the surface features.

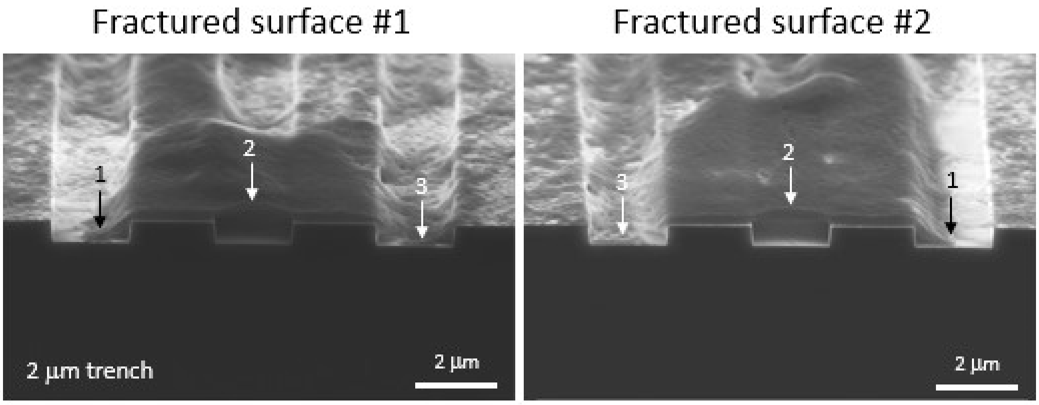

Figure S8. Tilted SEM micrographs of a cross-sectional cell adhered on the 2 µm trench patterned structure. Landmarks "1"–"3" are used to aid matching the surface features. Note the center portion of this nucleus bridges across the trench while the rest of the cell conformal coat the surface topography.

© 2018 by the authors. Submitted for possible open access publication under the terms and conditions of the Creative Commons Attribution (CC BY) license (http://creativecommons.org/licenses/by/4.0/).
